# *Helichrysum gymnocephalum* Essential Oil: Chemical Composition and Cytotoxic, Antimalarial and Antioxidant Activities, Attribution of the Activity Origin by Correlations

**DOI:** 10.3390/molecules16108273

**Published:** 2011-09-29

**Authors:** Samia Afoulous, Hicham Ferhout, Emmanuel Guy Raoelison, Alexis Valentin, Béatrice Moukarzel, François Couderc, Jalloul Bouajila

**Affiliations:** 1 Laboratoire des Interactions Moléculaires et Réactivité Chimique et Photochimique UMR CNRS 5623, University of Toulouse, University of Paul-Sabatier, 118 route de Narbonne, F-31062 Toulouse, France; 2 Nat’Ex Biotech. Bat 7, 55 avenue Louis Breguet, 31400 Toulouse, France; 3 Laboratoire de Phytochimie et Standardisation, IMRA, BP: 3833 Antananarivo 101, Madagascar; 4 Faculté of Pharmacy, University of Toulouse, UMR 152 IRD-UPS Pharma-DEV, University of Paul Sabatier Toulouse 3, 35 chemin des maraîchers, 31062 Toulouse Cedex 9, France

**Keywords:** *Helichrysum gymnocephalum*, GC-MS, antimalarial activity, cytotoxic activity, antioxidant activity

## Abstract

*Helichrysum gymnocephalum* essential oil (EO) was prepared by hydrodistillation of its leaves and characterized by GC-MS and quantified by GC-FID. Twenty three compounds were identified. 1,8-Cineole (47.4%), bicyclosesquiphellandrene (5.6%), γ-curcumene (5.6%), α-amorphene (5.1%) and bicyclogermacrene (5%) were the main components. Our results confirmed the important chemical variability of *H. gymnocephalum*. The essential oil was tested *in vitro* for cytotoxic (on human breast cancer cells MCF-7), antimalarial (*Plasmodium falciparum*: FcB1-Columbia strain, chloroquine-resistant) and antioxidant (ABTS and DPPH assays) activities. *H. gymnocephalum *EO was found to be active against MCF-7 cells, with an IC_50_ of 16 ± 2 mg/L. The essential oil was active against *P. falciparum* (IC_50_ = 25 ± 1 mg/L). However, the essential oil exhibited a poor antioxidant activity in the DPPH (IC_50_ value > 1,000 mg/L) and ABTS (IC_50_ value = 1,487.67 ± 47.70 mg/L) assays. We have reviewed the existing results on the anticancer activity of essential oils on MCF-7 cell line and on their antiplasmodial activity against the *P. falciparum*. The aim was to establish correlations between the identified compounds and their biological activities (antiplasmodial and anticancer). β-Selinene (R² = 0.76), α-terpinolene (R² = 0.88) and aromadendrene (R² = 0.90) presented a higher relationship with the anti-cancer activity. However, only calamenene (R² = 0.70) showed a significant correlation for the antiplasmodial activity.

## 1. Introduction

The flora of Madagascar is particularly rich, with a specific diversity and more importantly, it contains endemic species. In Madagascar, more than 400 species described in other parts of the World, especially in Southern Africa, Western Europe and Australia, and 115 species, all endemic of the large Island, are currently known [1]. The gender *Helichrysum* (from the Greek "helios" sun and "chrysos" gold) which belongs to Asteraceae family is one of the endemic species.

*Helichrysum gymnocephalum* (Immortal with naked head) is a shrub from 1 to 4 meters in height whose ultimate branches are covered by a white and very fine tomentum. The leaves are lengthily attenuated towards the base in short petioles, covered on the two faces by a tomentum and having three quite visible principal veins in lower part. Flowering lasts from February till May, seeds are also papillate [2].

Some medicinal properties of *Helichrysum* species have been reported, and various studies have demonstrated the antimicrobial and antiseptic properties of many species from the genus *Helichrysum* [3,4,5]. *H. gymnocephalum* has been traditionally used therapeutically as a tea or syrup prepared from the leaves to treat gingivitis or buccal ulcers [6]. It is also used as ointment for rheumatism or gout by mixing crushed leaves with bacon. The leaves and flowers have properties as diuretics, stimulants and would relieve neuralgia [7]. Analgesic, aphrodisiac, antiseptic, antiscorbutic, deodorant, tonic and anthelmintic applications have been reported for *H. gymnocephalum* [8,9]. *H. gymnocephalum* is also used in the manufacture of toothpaste for the local market. The leaves are also used to embalm dead bodies.

The essential oil (EO), a complex mixture of compounds, is considered among the most important antimicrobial agents [3] present in these plants, and may also have antioxidant and anti-inflammatory activities. These last years, an overflow of information has appeared on the role of the oxidative stress in the emergence of a certain number of diseases, such as cancers, cardiovascular diseases and degenerative diseases related to ageing, in parallel the possible therapeutic role of antioxidants in these diseases was emphasized [10].

More than a third of the World’s population (about two billion people) lives in malaria-endemic areas. The majority of deaths caused by falciparum malaria occur in sub-Saharan Africa, primarily among children younger than 5 years and pregnant women living in remote rural areas with limited access to health services [11]. Nearly all malaria deaths and a large proportion of morbidity are caused by *Plasmodium falciparum*. In the last decades, resistance to several antimalarial drugs became widely disseminated, while the cost of effective treatments was prohibitive for the majority of the populations in the endemic regions. Thus, rapid development of resistance by *P. falciparum* to the conventional drugs necessitates search for new antimalarial drugs [11].

Breast cancer is second only to lung cancer as the most common cancer in women. Roughly 180,000 women are diagnosed with this disease each year, of which 44,000 or almost 20% will die [12]. With increased awareness and increased use of routine mammograms, more women are diagnosed in the earlier stages of this disease, at which time a cure may be possible. The disease is more common in women after the age of 40. It is also more frequent in women of a higher social-economic class [12]. Cancer diseases are characterized by an uncontrolled proliferation of cells. They constitute the second cause of mortality behind cardiovascular diseases in developed countries and the third after infectious and cardiovascular diseases in developing countries [13]. The use of plant extracts and derived products in the treatment of cancer is of exceptional value in the control of malignancies, due to the fact that most of the anticancer drugs severely affect the normal cells [13,14].

The aim of the present study was the evaluation of *H. gymnocephalum* leaves essential oil for its possible antioxidant and biological activities. In this study, we: (i) examined the chemical components of the essential oil by GC-MS and GC-FID; (ii) evaluated their cytotoxic, antimalarial and antioxidant activities and (iii) reviewed researches for essential oils having an activity against *P. falciparum* and/or on MCF-7 cell line in order to identify, by correlation, the main active compounds.

## 2. Results and discussion

### 2.1. Chemical Composition

The essential oil yield of H. *Gymnocephalum* obtained from hydrodistillation of leaves was 0.40%. Two previous works have quantified the yield of this essential oil. They studied the shoot part of the plant mainly bark and leaves; extraction yields for essential oil were 0.5% and 0.41% according to Mollenbeck *et al*. [26] and Cavalli *et al.* [27], respectively. In the present work, only the leaves were used and a similar essential oil yield was obtained.

**Figure 1:**
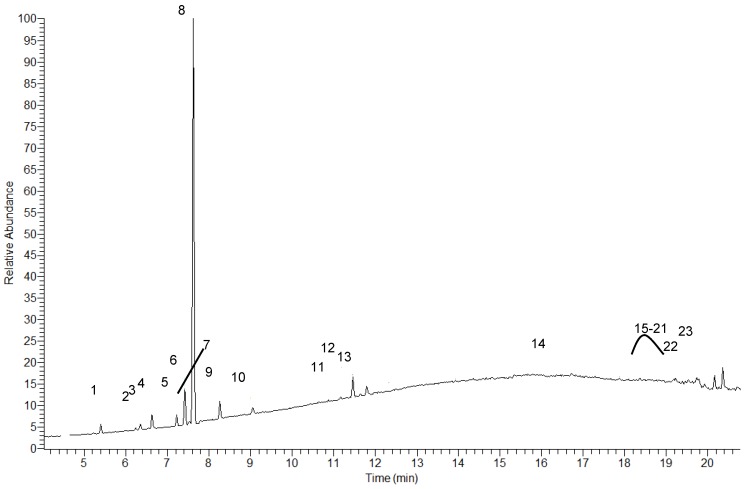
Chromatograms GC-MS of *H. gymnocephalum *essential oil (1: α-thujene; 2: sabinene; 3: β-pinene; 4: 2,3-dihydro-1,8-cineole; 5: α-terpinene; 6: *p*-cymene; 7: limonene; 8: 1,8-cineole; 9: (E)-β-ocimene; 10: α-terpinolene; 11: α-phellandrene; 12: terpinen-4-ol; 13: α-terpineol; 14: α-copaene; 15: aromadendrene; 16: bicyclo-sesquiphellandrene; 17: γ-curcumene; 18: β-selinene; 19: bicyclogermacrene; 20: α-amorphene; 21: 2,3-di-*tert*-butylphenol; 22: calamenene; 23: δ-cadinene).

Twenty three components have been identified in *H. gymnocephalum* essential oil by GC-MS (Figure 1 and Table 1). Sesquiterpene hydrocarbons and oxygenated monoterpenes were the major groups of compounds (32.4% and 54.0%, respectively), while oxygenated sesquiterpenes were not present. The major monoterpenes found were 1,8-cineole (47.4%), *p*-cymene (4.3%), (*E*)-β-ocimene (2.4%), 2,3-dihydro-1,8-cineole (2.1%) and α-terpinolene (1,3%). Among the sesquiterpene hydrocarbons bicyclosesquiphellandrene (5.6%), γ-curcumene (5.6%), α-amorphene (5.1%) and bicyclogermacrene (5%) were present in appreciable quantities. On the other hand, this essential oil contained a phenolic compound (2,3-di-*tert*-butylphenol) at 0.5%.

The chemical composition of *H. gymnocephalum* essential oil has been reported elsewhere. Mollenbeck *et al.* [26] have shown that 1,8-cineole (66.7%) was the major compound of the essential oil, a level which was higher than in our work (47.7%). Also, in our work, the major compounds were β-caryophyllene (3.3%), γ-terpinene (3.1%), β-pinene (2.7%), α-pinene (2.5%) and ρ-cymene (2.3%).

**Table 1:** Chemical composition of *H. gymnocephalum* essential oil.

N	RI	Compounds	(%)
**1**	928	α-Thujene	1.0
**2**	967	Sabinene	0.3
**3**	971	β-Pinene	1.1
**4**	984	2,3-Dihydro-1,8-cineole	2.1
**5**	1010	α-Terpinene	1.3
**6**	1018	*p*-Cymene	4.3
**7**	1022	Limonene	0.5
**8**	1026	1,8-Cineole	47.4
**9**	1052	(*E*)-β-Ocimene	2.4
**10**	1084	α-Terpinolene	1.3
**11**	1164	α-Phellandrene	0.2
**12**	1175	Terpinen-4-ol	2.7
**13**	1187	α-Terpineol	1.8
**14**	1373	α-Copaene	0.4
**15**	1438	Aromadendrene	2.0
**16**	1470	Bicyclosesquiphellandrene	5.6
**17**	1473	γ-Curcumene	5.6
**18**	1485	β-Selinene	3.3
**19**	1494	Bicyclogermacrene	5.0
**20**	1497	α-Amorphene	5.1
**21**	1502	2,3-di-*tert*-butylphenol	0.5
**22**	1512	Calamenene	1.8
**23**	1521	δ-Cadinene	3.6
Identified components			99.3
Monoterpene hydrocarbons			8.1
Monoterpenes oxygenated			54.0
Sesquiterpenes hydrocarbons			32.4
Others			4.8

Cavalli *et al.* [27] have shown that the chemical composition of the essential oil of *H. gymnocephalum* was dominated by 1,8-cineole (59.7%), *p*-cymene (6.3%), α-pinene (4.9%), β-pinene (3.3%) and terpinen-4-ol (2.6%). These published compositions of *H. gymnocephalum* essential oil indicated the presence of oxygenated sesquiterpenes, which were not present in our work. On the other hand, we found more α-thujene (1%) and δ-cadinene (3.6%) compared to Cavalli *et al*. [27] 0.5% and 0.5%, respectively. For terpinen-4-ol, we obtained 2.6% which was similar to the result of Cavalli *et al.* [27] (2.7%) but higher than the result of Mollenbeck *et al.* [26] (1.3%). An intermediate concentration (4.3%) of *p*-cymene was obtained compared to Cavalli *et al.* [27] and Mollenbeck *et al.* [26], with 6.3% and 2.3%, respectively. On the other hand, β-pinene (1.1%) was obtained in the lowest concentration compared to these two studies of Cavalli *et al*. [27] and Mollenbeck *et al*. [26] (3% and 2.7%, respectively).

**Figure 2:**
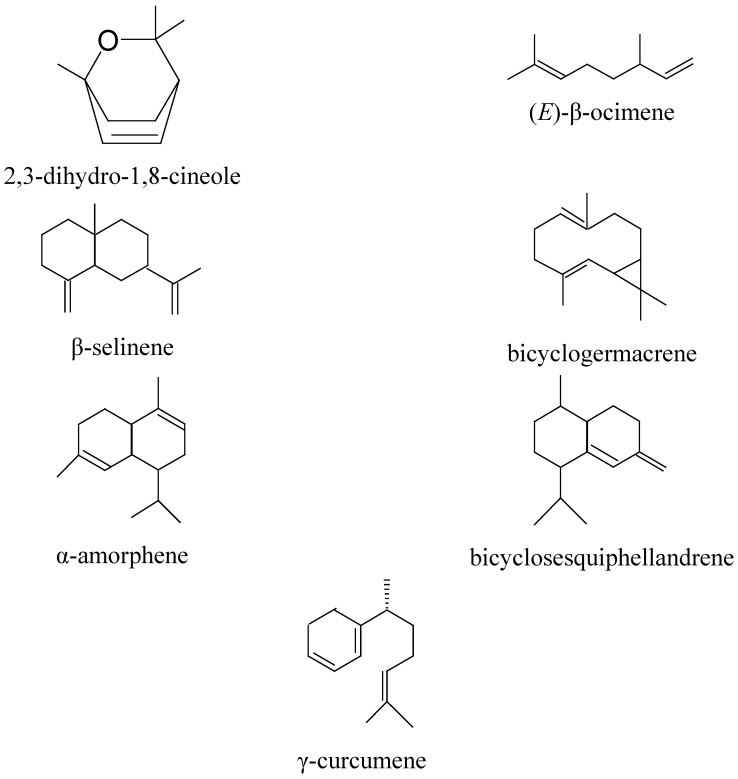
Structures of new abundant compounds identified in *H. gymnocephalum *essential oil compared to other studies of same essential oil.

We identified some new compounds which were not reported in these two previous studies. The total yield of these new compounds varied between 2.1 and 5.6% (Figure 2). The presence of 2,3-dihydro-1,8-cineole (2.1%), (*E*)-β-ocimene (2.4%), β-selinene (3.3%), bicyclogermacrene (5.0%), α-amorphene (5.1%), bicyclosesquiphellandrene (5.6%) and γ-curcumene (5.6%) was evidenced.

As shown in Table 1, the differences between our work and previously published results concerned extraction yield, the number of identified compounds and their respective contents. In our investigation, *H. gymnocephalum* plants were harvested in July 2008, and we treated only leaves for the essential oil extraction. Indeed, H. *gymnocephalum *was harvested in March 1997 by the team of Mollenbeck *et al*. [26] and between November–December 1994 by Cavalli *et al*. [27].

### 2.2. Antioxidant Activity

Data presented here is the first bibliographical report on the antioxidant activity of *H. gymnocephalum.* Furthermore, our results (Table 2) demonstrated that the essential oil of *H. gymnocephalum* has poor antioxidant activity against DPPH (IC_50_ value > 1,000 mg/L) and ABTS^+^ (IC_50_ = 1,487.67 ± 47.70 mg/L). These results may be attributed to a low antioxidant activity (in these two tests) of the compounds identified in the essential oil of *H. gymnocephalum.* Four other *Helichrysum* species (*H. dasyanthum*, *H. excisum*, *H. felinum* and *H. petiolare*) were also reported to exhibit also a low antioxidant activity against test DPPH (IC_50_ > 100 mg/L) [28].

**Table 2:** Anticancer, antimalarial and antioxidant activities (IC50 (mg/L)) of *H. gymnocephalum* essential oil.

Sample	Anticanceractivity	Antiplasmodial activity	Antioxidant activity (DPPH assay)	Antioxidant activity (ABTS assay)
Essential oil	16 ± 2	25 ± 1	>10000	1487.67±47.70
Control	0.218 ^a^ ± 0.04	0.10 ^b^ ± 0.09	3.75 ^c^ ± 0.01	1.84 ^c^ ± 0.03

^a^ Doxorubicin; ^b^ Chloroquine; ^c^ Ascorbic acid.

### 2.3. Cytotoxic Activity

In recent years, considerable attention has been focused to identifying naturally occurring substances able to inhibit, delay or reverse the process of multistage carcinogenesis. Plant essential oils are believed to reduce the risk of cancer when used in prevention [29].

In this work, the cytotoxic activity of *H. gymnocephalum* leaf essential oil against human breast cancer cells MCF-7 (IC_50_ = 16 ± 2 mg/L) was reported for the first time.

Data in Table 3 shows the anti-cancer activity against MCF-7 cells (IC_50_ mg/L) of several essential oils reported in the literature and their main components. Essential oils were extracted from *H. gymnocephalum*, *Schefflera heptaphylla *[30], *Laurus nobilis*, *Origanum syriacum*, *Origanum vulgare*, *Salvia triloba *[31], *Heteropyxis dehniae *[32], *Schinus molle*, *Schinus terebenthifolius *[33], *Salvia officinalis *[34], *Melaleuca alternifolia *[35], *Citrus limon*, *Citrus medica*, *Citrus sinensis *[36] *and Talauma gloriensis *[37]*.*

Based on the bibliographical review, we can consider that the IC_50_ of our essential oil (16 mg/L ± 2) is very well positioned among these studied oils in the Table 3. Among the studied essential oils only five showed higher anti-cancer activities than *H. gymnocephalum*’sessential oil. Indeed, nine essential oils had lower activities for the same test (Table 3).

### 2.4. Antimalarial Activity

Many studies on the antiplasmodial activity of crude essential oils have been reported [38,39]. The *in vitro *antiplasmodial activity of *H. gymnocephalum* essential oil was determined against the FcB1 chloroquine-resistant strain of *P. falciparum* (Table 2). The antimalarial activity of the essential oil (IC_50_ values) was 25 ± 1 mg/L. Since the value of IC_50_ was found between 5 and 50 mg/L, it can be considered that *H. gymnocephalum *essential oil has a good activity against *P. falciparum *[24]. This rather high value of IC_50_ compared to that of chloroquine (IC_50_ = 0.1 ± 0.09 mg/L) can be explained by the low concentration of the active compound(s) since the essential oil is a multi-components mixture.

Data in Table 4 summarize the antimalarial activity [IC_50_ (mg/L)] of all essential oils cited in the literature and their components. These EO have been obtained from *H. gymnocephalum*, *Xylopia phloiodora*, *Pachypodanthium conﬁne*, *Antidesma laciniatum*, *Xylopia aethiopica*, *Hexalobus crispiﬂorus* [40], *Salvia stenophylla*, *Salvia runcinata*, *Salvia repens *[41], *Salvia albicaulis*, *Salvia dolomitica *[42], *Lippia multiflora *[43], *Helichrysum cymosum *[44], *Artemisia gorgonum Webb *[45], *Arnica longifolia*, *Aster hesperius and Chrysothamnus nauseosus *[46]. The results based on this bibliographical review (Table 4) showed that the IC_50_ of our essential oil (25 mg/L ± 1) was at an interesting level, quite well positioned among these studied oils. We found seven essential oils which have higher antimalarial activity than that of *H. gymnocephalum *essential oil. In addition, 11 essential oils had lower activities in similar tests (Table 4).

**Table 3:** Anticancer activity and chemical composition of essential oils (I: *Helichrysum gymnocephalum*; II: *Schefflera heptaphylla *[30]; III: *Laurus nobilis*; IV: *Origanum syriacum*; V: *Origanum vulgare*; VI: *Salvia triloba *[31]; VII: *Heteropyxis dehniae *[32]; VIII: *Schinus molle*; IX: *Schinus terebenthifolius *[33]; X: *Salvia officinalis *[34]; XI: *Melaleuca alternifolia *[35]; XII: *Citrus limon*; XIII: *Citrus medica*; XIV: *Citrus sinensis *[36]; XV: *Talauma gloriensis *[37]).

**Essential oil**	**I**	**II**	**III**	**IV**	**V**	**VI**	**VII**	**VIII**	**IX**
**Anticancer activity (IC_50_ mg/L)**	16 ± 2	7.3	101.7 ± 7.9	130 ± 52.2	30.1 ± 1.14	174.3 ± 73.04	150 *	54 ± 10	47 ± 9
**Component**									
α-Thujene	1		0.62	0.47	0.54	0.38			
Sabinene	0.3		6.92	0.35	5.56	0.24		0.02	0.02
β-Pinene	1	22.24	4.55	0.6	1.3	8.89		4.96	3.09
2,3-Dihydro-1,8-cineole	2								
α-Terpinene	1.2		0.7	0.37	4.46	0.44			
*p*-Cymene	4.2		0.74	30.22	3.83	0.34	1.5	2.49	7.34
Limonene	0.5	3.61	2.1		1.68		0.9		
1,8-Cineole	47.4		40.91	0.27		45.16	0.4		
(*E*)-β-Ocimene	2.4								
α-Terpinolene	1.3			0.35		0.43			
α-Phellandrene	0.2							46.52	34.38
Terpinen-4-ol	2.7		1.55			1.09		0.07	0.03
α-Terpineol	1.8					0.62	3.6	8.38	5.6
α-Copaene	0.4						0.6	0.11	0.19
Aromadendrene	2							0.49	0.19
Bicyclosesquiphellandrene	5.6								
γ-Curcumene	5.6								
β-Selinene	3.3						0.3	1.1	
Bicyclogermacrene	5				1.05				
α-Amorphene	5.1								
2,3-Di-*tert*-butylphenol	0.5								
Calamenene	1.8								
δ-Cadinene	3.6						2.8	0.27	0.69
**Essential oil**	**X**	**XI**	**XII**	**XIII**	**XIV**	**XV**			
						[37]			
**Anticancer activity (IC_50_ mg/L)**	554.4 ± 1.5	310 *	10	1	0.5	14.1			
**Component**									
α-Thujene	0.31								
Sabinene	0.41				0.1				
β-Pinene	2.57	0.91		16.3		3.7			
2,3-Dihydro-1,8-cineole									
α-Terpinene	0.2	5.76				0.1			
*p*-Cymene									
Limonene	1.7		98.4	56.6	98.4	0.8			
1,8-Cineole	17.52	19.29							
(*E*)-β-Ocimene						0.1			
α-Terpinolene									
α-Phellandrene									
Terpinen-4-ol	1.01	42.62							
α-Terpineol	0.27			11.3					
α-Copaene						0.1			
Aromadendrene									
Bicyclosesquiphellandrene									
γ-Curcumene									
β-Selinene									
Bicyclogermacrene						2.1			
α-Amorphene									
2,3-Di-*tert*-butylphenol									
Calamenene									
δ-Cadinene						3.3			

**Table 4:** Antipaludic activity and chemical composition of essential oils (I: *Helichrysum gymnocephalum*; II: *Xylopia phloiodora*; III: *Pachypodanthium conﬁne*; IV: *Antidesma laciniatum*; V: *Xylopia aethiopica*; VI: *Hexalobus crispiﬂorus* [40]; VII: *Salvia stenophylla*; VIII: *Salvia runcinata*; IX: *Salvia repens *[41]; X: *Salvia albicaulis*; XI: *Salvia dolomitica* [42]; XII: *Lippia multiflora *[43]; XIII: *Helichrysum cymosum *[44]; XIV: *Artemisia gorgonum Webb* [45]; XV: *Arnica longifolia*; XVI: *Aster hesperius*; XVII: *Chrysothamnus nauseosus *[46]; XVIII: Essential oil [39]). * IC_50_ was calculated from percentage; na: no activity.

**Essential oil**	**I**	**II**	**III**	**IV**	**V**	**VI**	**VII**	**VIII**	**IX**	**X**	**XI**
**Antipalaudic activity (IC_50_ mg/L)**	25 ± 1	17.9	16.6	29.4	17.8	2.0	4.38 ± 1.07	1.23 ± 0.31	1.68 ± 0.26	6.4 ± 2.0	4.8 ± 0.7
**Component **											
α-Thujene	1.0		0.59		0.61						
Sabinene	0.3		0.59		0.46		0.1		0.2		0.1
β-Pinene	1.0	0.68			10.07	0.12	0.7	0.8	3.0		
2,3-Dihydro-1,8-cineole	2.0										
α-Terpinene	1.2		0.48		0.43		0.3		0.2		
*p*-Cymene	4.2	0.35	0.32		1.72			0.2	0.7	2.5	0.2
Limonene	0.5						5.3	0.6	9.8	9.4	0.6
1,8-Cineole	47.4							2.0		9.4	
(*E*)-β-Ocimene	2.4		1.93		1.13			0.8	1.5		
α-Terpinolene	1.3										
α-Phellandrene	0.2										
Terpinen-4-ol	2.7		0.16		0.49	0.16					0.8
α-Terpineol	1.8				4.99					2.7	6.2
α-Copaene	0.4	0.53	7.06	2.2	4.07	13.27		0.1			
Aromadendrene	2.0					1.08				2	
Bicyclosesquiphellandrene	5.6										
γ-Curcumene	5.6										
β-Selinene	3.3	0.28				2.2					
Bicyclogermacrene	5.0										
α-Amorphene	5.1										
2,3-Di-*tert*-butylphenol	0.5										
Calamenene	1.8		2.32		0.93	1.09					
δ-Cadinene	3.6	15.11	8.06	1.3	4.3	10.07	0.5		0.3		
**Essential oil**	**XII**	**XIII**	**XIV**	**XV**	**XVI**	**XVII**	**XVIII**				
**Antipalaudic activity (IC_50_ mg/L)**	2–4	1.25 ± 0.77	5.2 ± 0.77	na	na	na	40.15 *	205.19 *	10.82 *	72.68 *	
**Component **											
α-Thujene				0.8		0.8					
Sabinene	0.3	0.2									
β-Pinene		3.7					96.32				
2,3-Dihydro-1,8-cineole											
α-Terpinene		1.2				1.0					
*p*-Cymene	0.2	2.4		0.1	0.1	0.6		93.19			
Limonene	0.2	7.2				4.7				92.98	
1,8-Cineole	3.1	20.4		0.1	0.1				93.13		
(*E*)-β-Ocimene		3.6				0.3					
α-Terpinolene											
α-Phellandrene	1.8										
Terpinen-4-ol			0.2								
α-Terpineol	0.3	2.6	0.2	0.2	0.1	0.3					
α-Copaene	0.3	1.2			0.2						
Aromadendrene		1.5									
Bicyclosesquiphellandrene											
γ-Curcumene			0.5								
β-Selinene			0.3								
Bicyclogermacrene											
α-Amorphene											
2,3-Di-*tert*-butylphenol											
Calamenene					0.1	0.2					
δ-Cadinene	0.2			0.4	0.8						

To study the role of the various components of an essential oil in the biological activities obtained, we performed a complete survey of the activities and the percentage of compounds present in order to assign the origin of the activity. The aim of the study was to find correlations between each component present in our literature search of all essential oils of various plants tested for biological activities (anticancer and antimalarial). Correlations between our essential oil, other oils and their components are listed in Table 3 and Table 4, with the IC_50_ on the tested biological target (MCF-7 cell line or *P. falciparum*).

### 2.5. Cytotoxic Activity Correlations

**Figure 3:**
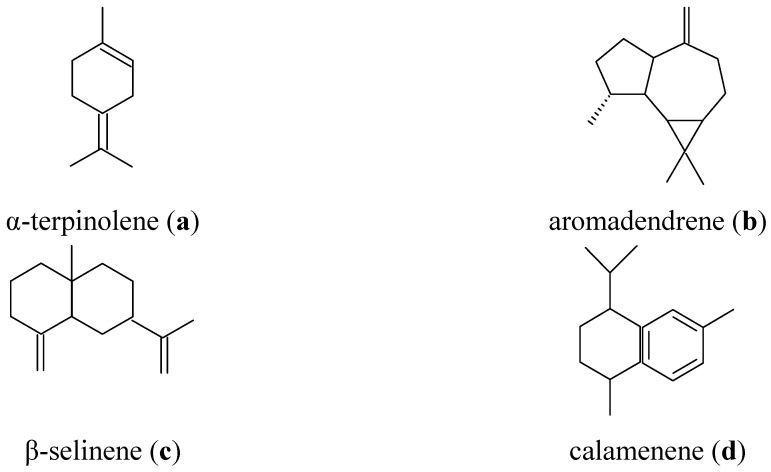
Structures of compounds which have good correlation for anticancer (**a–c**) and antimalarial (**d**) activities.

We established correlations between compound contents and anti-cancer activity against the MCF-7 cells. Aromadendrene, α-terpinolene and β-selinene showed good correlations, respectively R² = 0.90, 0.88 and 0.76 (chemical structures are presented in Figure 3).

To our knowledge, no literature has cited any anticancer activity of these three compounds (β-selinene, aromadendrene and α-terpinolene). We will carry out a deeper study to focus on their specific biological activity against MCF-7 cell line.

Based on this correlation study, we noticed that six components were present only in our essential oil, namely bicyclosesquiphellandrene (5.6%), α-amorphene (5.1%), 2,3-dihydro-1,8-cineole (2%), limonene (0.5%), and 2,3-di-*tert*-butylphenol (0.5%). In addition, for bicyclogermacrene (5.0%) and (*E*)-β-ocimene (2.4%), the correlation has not been established because we did not have enough data (only two essential oils).

A deep study of the anticancer activity of aromadendrene, α-terpinolene, β-selinene, bicyclo-sesquiphellandrene, α-amorphene, 2,3-dihydro-1,8-cineole, calamenene, 2,3-di-*tert*-butylphenol, bicyclogermacrene and (*E*)-β-ocimene will have to be carried out to determine the origin of the anticancer activity of *H. gymnocephalum*. On the same target, we should evaluate the synergy between these compounds and their own contribution to the final anticancer activity.

### 2.6. Correlations for Antimalarial Activity

We established correlations between both major and minor components and IC_50_ (antimalarial activity). Calamenene showed the highest correlation R^2^ = 0.7 (Figure 3). Our results are in accordance with those of Van Zyl *et al.* [39]; the former showed that *p*-cymene, limonene and β-pinene had low antiplasmodial activity (IC_50_ = 205.20, 72 and 40.16 mg/L, respectively). Indeed, they also showed that there are significant synergies (the sum of the fractional inhibitory concentrations (∑FIC) < 0.5) between *p*-cymene and other compounds such as *E-* and *Z*-(±)-nerolidol, carvacrol and γ-terpinene (0.09, 0.02 and 0.37, respectively). We noticed that bicyclosesquiphellandrene (5.6%), α-amorphene (5.1%), bicyclogermacrene (5.0%), 2,3-dihydro-1,8-cineole (2.0%), and 2,3-di-*tert*-butyl-phenol (0.5%) were present only in our essential oil. It is possible that these compounds may be involved in the activity of our essential oil.

## 3. Experimental

### 3.1. Extraction of the Essential Oil

The leaves of *H. gymnocephalum* were collected in Antananarivo, Madagascar (July 2008). Steam-distillation was used to extract the essential oil according to the European Pharmacopoeia protocol [15]. The essential oil was dried with anhydrous sodium sulphate, filtered and stored in sealed vials at 4 °C prior to analyses.

### 3.2. Chemicals

All chemicals used were of analytical reagent grade. All reagents were purchased from Sigma- Aldrich-Fluka (Saint-Quentin, France).

### 3.3. Gas Chromatography and Gas Chromatography-Mass Spectrometry

Quantitative and qualitative analysis of the essential oil was carried out by gas chromatography-flame ionization detection (GC-FID) and gas chromatography-mass spectrometry (GC-MS). Gas chromatography analyses were carried out on a Varian Star 3400 Cx chromatograph (Les Ulis, France) fitted with a fused silica capillary DB-5MS column (5% phenylmethylpolysyloxane, 30 m × 0.25 mm, film thickness 0.25 µm). Chromatographic conditions were 60 °C to 260 °C temperature rise with a gradient of 5 °C/min and 15 min isotherm at 260 °C. A second gradient was applied to 340 °C at 40 °C/min. Total analysis time was 57 min. For analysis purposes, the essential oil was dissolved in petroleum ether. One microliter of sample was injected in the split mode ratio of 1:10. Helium (purity 99.999%) was used as carrier gas at 1 mL/min. The injector was operated at 200 °C. The mass spectrometer (Varian Saturn GC/MS/MS 4D) was adjusted for an emission current of 10 µA and electron multiplier voltage between 1,400 and 1,500 V. Trap temperature was 150 °C and that of the transfer line was 170 °C. Mass scanning was from 40 to 650 amu.

Compounds were identified by comparison of their Kovats indices (KI) obtained on a nonpolar DB-5MS column relative to C_5_-C_24_* n*-alkanes, with those provided in the literature, by comparison of their mass spectra with those recorded in NIST 08 (National Institute of Standards and Technology) and reported in published articles and by co-injection of available reference compounds. The samples were analyzed in duplicate. The percentage composition of the essential oil was computed by the normalization method from the GC peak areas, assuming identical mass response factors for all compounds. Results were calculated as mean values of two injections from essential oil, without using correction factors. All determinations were performed in triplicate and averaged.

### 3.4. Antioxidant Activity

#### Free Radical Scavenging Activity: DPPH Test

Antioxidant scavenging activity was studied using the 1,1-diphenyl-2-picrylhydrazyl free radical (DPPH) assay as described by Blois [16] with some modifications. Various dilutions of EO (1.5 mL) were mixed with a 0.2 mmol/L methanolic DPPH solution (1.5 mL). After an incubation period of 30 min at 25 °C, the absorbance at 520 nm (the wavelength of maximum absorbance of DPPH) were recorded as A_(sample)_, using a Helios spectrophotometer (Unicam, Cambridge, UK). A blank experiment was also carried out applying the same procedure to a solution without the test material and the absorbance was recorded as A_(blank)_. The free radical-scavenging activity of each solution was then calculated as percent inhibition according to the following equation:

% inhibition = 100 (A_(blank)_ − A_(sample)_) / A_(blank)_

Antioxidant activities of test compounds or the essential oil were expressed as IC_50_ values, defined as the concentration of the test material required to cause a 50% decrease in initial DPPH concentration. Ascorbic acid was used as a standard. All measurements were performed in triplicate.

### 3.5. ABTS Radical-Scavenging Assay

The radical scavenging capacity of the samples for the ABTS (2,2′-azinobis-3-ethylbenzothiazoline-6-sulphonate) radical cation was determined as described by Re *et al*. [17] with some modifications. ABTS was generated by mixing a 7 mmol/L solution of ABTS at pH 7.4 (5 mmol/L NaH_2_PO_4_, 5 mmol/L Na_2_HPO_4 _and 154 mmol/L NaCl) with 2.5 mmol/L potassium persulfate (final concentration) followed by storage in the dark at room temperature for 16 h before use. The mixture was diluted with ethanol to give an absorbance of 0.70 ± 0.02 units at 734 nm using a spectrophotometer (Helios). For samples, solutions of the essential oil in methanol (100 μL) were allowed to react with fresh ABTS solution (900 μL), and then the absorbance was measured 6 min after initial mixing. Ascorbic acid was used as a standard and the capacity of free radical scavenging was expressed by IC_50_ (mg/L). IC_50_ values were calculated as the concentration required for scavenging 50% of ABTS radicals. The capacity of free radical scavenging (IC_50_) was determined using the same previously used equation for the DPPH method. All measurements were performed in triplicate. All data of antioxidant activity were expressed as means ± standard deviations (SD) of the triplicate measurements. The confidence limits were set at P < 0.05. SD did not exceed 5% for the majority of the values obtained.

### 3.6. Antiplasmodial Activity

The chloroquine-resistant FcB1-Columbia strain of *Plasmodium falciparum* (IC_50_ for chloroquine: 186 nM) was cultured continuously according to Trager and Jensen [18], with modifications [19]. The IC_50_ values for chloroquine were checked every 2 months, and we observed no significant variations. The parasites were maintained *in vitro* in human red blood cells (O^±^; EFS; Toulouse, France), diluted to 4% hematocrit in RPMI 1640 medium (Lonza, Emerainville, France) supplemented with 25 mM Hepes and 30 M NaHCO_3_ and complemented with 7% human AB^+^ serum (EFS).

Parasite cultures were synchronized by combination of magnetic enrichment [20] followed by D-sorbitol lysis (5% of D-sorbitol in sterile water) as described by Lambros and Vanderberg [21]. The antimalarial activity of essential oil was evaluated by a radioactive micromethod described elsewhere [22]. Tests were performed in triplicate in 96-well culture plates (TPP) with cultures mostly at ring stages (synchronisation interval, 16 h) at 0.5–1% parasitemia (hematocrit, 1.5%). Parasite culture was incubated with each sample for 48 h. Parasite growth was estimated by [^3^H]-hypoxanthine (Perkin-Elmer, Courtaboeuf, France) incorporation, which was added to the plates 24 h before freezing. After 48 h incubation, plates were frozen-defrosted and each well was harvested on a glass fiber filter. Incorporated (^3^H)-hypoxanthine was then determined with a β-counter (1450-Microbeta Trilux, Wallac-Perkin Elmer). The control parasite cultures, free from any sample, was referred to 100% growth. IC_50_ were determined graphically in concentration versus percent inhibition curves. Chloroquine diphosphate was used as positive control. The antimalarial activity of sample was expressed by IC_50_, representing the concentration of drug that induced a 50% parasitaemia decrease compared to the positive control culture referred to as 100% parasitaemia [23]. According to the literature concerning plant antiplasmodial activities a sample is very active if IC_50_ < 5 mg/L, active if IC_50_ between 5 and 50 mg/L, weakly active if IC_50_ between 50 and 100 mg/L and inactive if IC_50_ > 100 mg/L [24].

### 3.7. Cytotoxicity Evaluation

Cytotoxicity of sample was estimated on human breast cancer cells (MCF-7). The cells were cultured in the same conditions as those used for *P. falciparum*, except for the 10% human serum, which was replaced by 10% foetal calf serum (Lonza). For the determination of pure compound activity, cells were distributed in 96-well plates at 3 × 10^4^ cells/well in 100 µL, and then 100 µL of culture medium containing sample at various concentrations were added. Cell growth was estimated by (^3^H)-hypoxanthine incorporation after 48h incubation exactly as for the *P. falciparum* assay. The (^3^H)-hypoxanthine incorporation in the presence of sample was compared with that of control cultures without sample (positive control being doxorubicin) [25].

### 3.8. Statistical Analysis

All data were expressed as mean ± standard deviation of triplicate measurements. The confidence limits were set at P < 0.05. Standard deviations (SD) did not exceed 5% for the majority of the values obtained.

## 4. Conclusions

In conclusion, we have identified all volatile constituents of *H. gymnocephalum* leaf essential oil and evaluated its anticancer, antimalarial, and antioxidant activities. Our results clearly showed that this essential oil was active against the tumor cell lines MCF-7 and the FcB1 strain of *P. falciparum. *Based on established correlations, compounds such as α-terpinolene, aromadendrene and β-selinene against MCF-7, could be the best candidates for further analysis. Purification of these compounds is under development to test them separately. In addition, an in depth study could determine the mechanisms by which these compounds exert their biological activities. The results presented here can be considered as the first information on the anticancer and antimalarial properties of *H. gymnocephalum*. This may also contribute to our knowledge of the genus *Helichrysum*. These *in vitro* results provide some scientific validation for the widespread use of plants from this genus in traditional medicine.
